# Effectiveness of Nutritional Education for Obese Older Adults With Frailty: A Systematic Review and Meta-Analysis

**DOI:** 10.1093/geroni/igaa057.1291

**Published:** 2020-12-16

**Authors:** Yue-Heng Yin, Liu Yat Justina

**Affiliations:** 1 The Hong Kong Polytechnic University, Hong Kong, China; 2 School of Nursing , The Hong Kong Polytechnic University, Hong Kong, China

## Abstract

Obesity has been shown to intensify the decline of physical function and lead to frailty. Nutrition is an important method in managing obesity and frailty, while seldom reviews have ever explored the effects of nutritional education interventions. We conducted a systematic review (PROSPERO: CRD42019142403) to explore the effectiveness of nutritional education interventions in managing body composition and physio-psychosocial parameters related to frailty. Randomized controlled trials and quasi-experimental studies were searched in CINAHL, Cochrane Library, EMBASE, MEDLINE, PsycINFO, PubMed and Scopus from 2001 to 2019. Hand search for the reference lists of included papers was conducted as well. We assessed the quality of included studies by Cochrane risk of bias tool. Meta-analyses and narrative synthesis were used to analyse the data. Two studies with low risk of bias were screened from 180 articles, which involved 177 older people with an average age of 69.69±4.08 years old. The results showed that nutritional education was significantly effective in reducing body weight and fat mass than exercises, and it was beneficial to enhancing physical function and psychosocial well-being. But the effects of nutritional education in increasing muscle strength were not better than exercises. The combined effects of nutritional education and exercises were superior than either exercises or nutritional education interventions solely in preventing the loss of lean mass and bone marrow density, and in improving physical function. Due to limited numbers of relevant studies, the strong evidence of effectiveness of nutritional education interventions on reversing frailty is still lacking.

